# The Causes and Treatment of Chloroform Syncope

**Published:** 1897-05-01

**Authors:** 


### THE CAUSES AND TREATMENT OF CHLOROFORM SYNCOPE.

In a very able address, delivered before the Society of Anæsthetists, Mr. Leonard Hill has pointed out that death from chloroform may arise from two very different conditions. The main object of his remarks was no doubt to controvert the "pernicious and dangerous doctrine" put forward by the Hyderabad Commission that chloroform kills by paralysing the respiratory centre.

What, however, is likely to be of most interest to practical men is the very definite pronouncement as to what are, in Mr. Hill's opinion, the causes of the fatality of chloroform, and what is the treatment to be adopted in cases of "chloroform syncope." If we recognise that there is not enough blood in the body to fill the vascular system, and that it is only by the maintenance of a certain degree of vaso-motor tone that the various organs are supplied with a due amount of nutrient fluid, we see at once that anything which could abolish this mechanism of vaso-motor tone, this compensation for the effect of gravitation, and permit the blood either to sink to the lower portions of the body or accumulate in the abdominal viscera, would have the effect of starving the respiratory and other nervous centres, and ultimately abolishing the circulation altogether. Now shock is one of the influences by which this condition of affairs is induced; chloroform is another, and of all known agencies the most potent. The effect of a gradually-increased overdose of chloroform is, then, to paralyse the vaso-motor tone of the splanchnic and other areas and thus lower the vascular tension. No blood arrives at the heart, and therefore no blood leaves it, and although death may in such cases take place by failure of respiration, this failure is the result of a preceding failure of the vaso-motor mechanism on which the nutrition of the respiratory centre depends.

But, in addition to this mode of death, cessation of the circulation may occur from direct paralysis of the heart. It is now some years since it was shown by MacWilliam that the constant effect of chloroform was to cause a paralytic dilatation of the cardiac ventricles, and Mr. Leonard Hill says that he has verified his experiments over and over again, and has seen that, even when low percentages of chloroform are inhaled, the effect is to cause cardiac dilatation. What happens in too large a number of cases, as is shown by the records which we have recently published of deaths under chloroform, is that the chloroform directly paralyses the heart muscle and produces what is spoken of as paralysis in diastole. The sequence of events is well described by Mr. Hill.

"Concentrated vapour of chloroform is applied to the respiratory orifice, the nerve endings of the vagus in the respiratory tract are powerfully excited. The animal struggles, the glottis is closed, and by the violent contraction of the muscles the intrathoracic pressure is raised. . . . The effect of raising intrathoracic pressure is to diminish the output from the right heart, congest the venous system, and lower the arterial tension; the lungs are also compressed, and, to a large extent, are emptied of blood. Blood supply to the coronary arteries is diminished; . . . the oxygen in the blood is decreased owing to the prolonged holding of the breath. By these means the nutrition of the heart is impaired. Finally, on account of the excitation of the respiratory centre caused by the asphyxial blood, the animal is forced to take two or three deep inspirations. The lungs are immediately surcharged with chloroform vapour, and the blood reaches the coronary artery carrying a dose of chloroform sufficient to throw the heart into paralytic dilatation." Nothing could more accurately describe the sequence of events in too many cases of "chloroform syncope."

What, then, about treatment? First, in regard to the prevention of accidents, Mr. Hill says: "The inhaler should only be applied when the respiration is quiet, should be removed entirely if the patient show any sign of struggling. If this precaution be always taken, deaths from chloroform would become far more rare; nevertheless, it must always be looked upon by the inexperienced as a most dangerous drug, and one which should be avoided whenever ether can be appropriately substituted." When, however, syncope has taken place, the treatment will vary according to the manner of its occurrence. When it arises during prolonged anæsthesia, and the primary mischief lies in the anæmia of the bulb caused by the general vaso-motor paralysis, artificial respiration with the assumption of the horizontal position will always resuscitate the patient it it be applied in time. When, however, the heart is thrown into paralytic dilatation during primary anæsthetisation, or when after holding his breath the patient takes a deep inspiration and then the heart is found to have ceased beating, artificial respiration with the patient in the horizontal position is again the proper treatment, in addition to which "the heart should be rhythmically compressed by squeezing the thorax. If this does not quickly renew the pulse, the patient should be put into the vertical (feet downward) posture. The dilated heart is thereby completely and easily emptied of blood. Artificial respiration is maintained during this manœuvre, and the patient is brought once more into the horizontal posture. By rhythmic compression of the chest an efficient circulation is maintained through the coronary arteries; by first emptying and then filling the heart, a fresh supply of blood is brought into that organ. If this does not prove successful on the first trial it can be repeated."

It must be always remembered that however useful inversion or pressure on the abdomen may be in those cases which are due to interference with vaso-motor tone, they would but increase the paralytic dilatation of the heart. The heart in such cases wants emptying, not filling, and either of these modes of treatment would be worse than useless in this form of syncope.

The points to remember, then, are to remove the chloroform entirely whenever there is struggling or holding of breath, and if syncope occur, to use artificial respiration in the horizontal posture, together with rhythmic compression of the heart, and, if the syncope has come on in the early stage of administration or after a struggle, to raise the patient up for a moment in the course of the artificial respiration, so as to ensure the emptying of the over-distended cavities of the heart.

